# Temporary Interference over the Posterior Parietal Cortices Disrupts Thermoregulatory Control in Humans

**DOI:** 10.1371/journal.pone.0088209

**Published:** 2014-03-12

**Authors:** Alberto Gallace, Giovanna Soravia, Zaira Cattaneo, G. Lorimer Moseley, Giuseppe Vallar

**Affiliations:** 1 Department of Psychology, University of Milano-Bicocca, Milan, Italy; 2 The Sansom Institute for Health Research, University of South Australia, Adelaide, Australia; 3 Neuroscience Research Australia, Sydney, Australia; 4 IRCCS Istituto Auxologico Italiano, Milano, Italy; University of Reading, United Kingdom

## Abstract

The suggestion has recently been made that certain higher-order cortical areas involved in supporting multisensory representations of the body, and of the space around it, might also play a role in controlling thermoregulatory functions. Here we demonstrate that temporary interference with the function of one of these areas, the posterior parietal cortex, by repetitive transcranial magnetic stimulation, results in a decrease in limb temperature. By contrast, interference with the activity of a sensory-specific area (the primary somatosensory cortex) had no effect on temperature. The results of this experiment suggest that associative multisensory brain areas might exert a top-down modulation over basic physiological control. Such a function might be part of a larger neural circuit responsible for maintaining the integrity of the body at both a homeostatic and a psychological level.

## Introduction

A series of studies in the last few years has started to highlight the consequences of higher-order cognitive manipulations on the lower-level mechanisms involved in controlling physiological life-supporting functions [1], [2], see also [3]. In fact, recent findings suggest that psychological illusions regarding the ownership of our body can modulate thermoregulatory [1] and immune [4] system control in neurologically unimpaired participants [5]. For example, eliciting the illusion that a rubber hand is part of a person's body results in a reduction of skin temperature on the hand that is ‘replaced’ by the artificial one, and in a shift in the perceived temporal order of pairs of tactile stimuli, such that stimuli from the experimental hand are given less weighting (in terms of attentional/processing resources) than stimuli from the other hand [1], [5], [6]. Similarly, researchers have shown in patients with complex regional pain syndrome (CRPS), that changing the position of the affected limb in space can shift from pathological to near-normal the temperature of the affected limb [6], [7], and that this effect depends on the perceived location of the limb, not its true location or alignment [8].

It is important to note that CRPS may occur after stroke involving the posterior parietal cortex [9] and after peripheral limb trauma without stroke. The likelihood of developing CRPS after peripheral trauma is not affected by the severity of the injury, (see [10] for a review). Despite their very different aetiologies, the similarities between post-stroke and post-peripheral trauma CRPS are remarkable, (see [11] for a review). All of these similarities, combined with the recent discovery that cooling of the affected limb in CRPS is positively related to a spatially-defined shift in tactile processing [6], would seem to suggest that the processing of spatial variables occurring in higher-order brain areas, can have an important effect on thermoregulatory control.

In order to account for the rather unexpected findings coming from the study of CRPS patients, as well as for those from bodily illusions in neurologically normal participants, it has recently been suggested that a network of brain areas, named the ‘body matrix’, might be responsible for maintaining the integrity of the body at both the homeostatic (i.e., thermoregulation) and psychological levels [5]. This neural network might therefore supervise the distribution of cognitive and physiological resources necessary to protect the body surface and the space around it (cf. [12] for the concept of “neuromatrix”, a network of brain areas responsible for the subjective experience of pain). Within this network a key role is thought to be played by higher-order (multisensory) areas, such as the posterior parietal cortex (PPC). The PPC is responsible for integrating different (somatotopic and spatial) frames of reference used for the localization of external stimuli and of the position of the body in space, as well as for sustaining a coherent multisensory representation of the body [13]. Following on from these considerations, one might predict that modulation of activity in the PPC may have consequences for homeostatic functions, such as thermoregulatory control.

A number of studies on neurological patients have shown a relationship between brain damage and thermoregulatory control [14]–[15]. In particular, Naver, Blomstrand, Ekholm, Jensen, Karlsson, and Wallin [16] studied the incidence of symptoms of autonomic dysfunction after the acute phase of stroke. They found that 43% of the patients observed reported a sensation of coldness in the side of the body contralateral to the side of the lesion (contralesional). Moreover, basal skin blood flow and temperature were generally lower in the contralesional side of the patients' bodies. Note, however, that in this study no correlation between the area or size of the lesion and the thermoregulatory dysfunction was reported. Such work was later extended by Riedl, Beckmann, Neundorfer, Handwerker, and Birklein [17] to directly compare autonomic dysfunction in the first few days after stroke to that observed in chronic CRPS, using detailed neurological examination, thermal imaging and evaporation hygrometry. The authors described the two conditions as remarkably similar, leading to the suggestion that autonomic dysfunction have a central origin in the neural system. It is important to note, however, that both studies found no correlation between the area (and side [17]) of the lesion and autonomic control.

As far as a more accurate localization of the brain areas involved in thermoregulatory control is concerned, Ishii, Ohtsuki, Tamaoka, Mizusawa, and Shoji [18] reported two cases of reduced limb temperature following damage to the somatosensory cortex. However, it is worth noting that the presence of subcortical damage and of possible impairment of peripheral nerves in both of these patients undermine any strong conclusion about the causal role of the somatosensory cortex in thermoregulatory control. Satoh, Terada, Onouchi, Takeda, and Kuzuhara [19] also described a patient with somatosensory dysfunction after infarction of the left postcentral gyrus, limited to Brodmann's areas 1 and 2, who showed a decrease in the skin temperature of the right hand. On the basis of this observation, the authors concluded that skin temperature might be controlled somatotopically in the somatosensory cortex, but that further studies were needed in order to clarify the mechanism and the anatomic localization of skin temperature control [19]. To our knowledge, there are no subsequent reports on the possible involvement of the somatosensory cortex on thermoregulatory control in humans (see [20], for the observation of a modulation of body temperature after stimulation of the orbital frontal neocortex in anesthetized rats). In particular, the question of whether or not higher-order cortical areas can modulate the functioning of lower-level physiological mechanisms (as suggested by behavioral evidence; see [5]), still remains unanswered. The present study aimed to clarify this important issue by measuring the effect on body temperature of temporarily interfering with the function of the PPC or S1 using repetitive transcranic magnetic Stimulation (rTMS).

rTMS is a non-invasive technique of brain stimulation that can influence the brain's electrical activity by a pulsed magnetic field. The magnetic field is generated by passing brief current pulses through a coil of wire. An important aspect of rTMS is that the effects of each single pulse can summate with repeated application, leading to effects outlasting a stimulation session [21], [22] and usually to an interference with the stimulated area [22]–[25].

In order to better explore the neural mechanisms of ‘higher order’ thermoregulation (i.e., thermoregulatory functions mediated by multisensory associative areas) we used rTMS to interfere with the functioning of PPC or S1. Following on from the body matrix theory [5] highlighted above, our primary hypothesis was that rTMS over PPC (and in particular over the right PPC) would modulate body temperature, but rTMS over S1 would not. Given that the right PPC has been shown to be involved in disorders of body ownership [26], one might expect that the stimulation of this area results in a modulation of body ownership as well as in changes of thermoregulatory control. Finally, considering that previous experiments where bodily illusions were adopted in order to induce changes of thermoregulation have found body-specific effects (e.g., inducing the illusion that the right hand is replaced by a rubber hand results in a reduction of the temperature in the real participant's right hand but not on his/her left hand) one might also expect a body specific modulation of thermoregulatory control following the stimulation of different brain hemispheres.

## Methods

### Participants

Twenty-four healthy volunteers (12 females) participated in the experiment. Their ages ranged from 20 to 30 years (mean ± S. D. = 24.3±2.5 years). The participants were right-handed, as assessed by a 10-item questionnaire [27], and had no psychiatric, neurological or other relevant medical disorders, as well as no contraindication to TMS [28]. The volunteers gave written informed consent and received course credits in return for their participation. The study reported in this paper was approved by the ethical committee of University of Milano-Bicocca. The experiment was performed in accordance with the ethical standards laid down in the 1991 Declaration of Helsinki.

### Materials

Two 17.5 mm×17.5 mm×6 mm ‘Thermocron-TC’ temperature loggers were used in order to constantly monitor and record the participant's body temperature with an accuracy of 0.07°C (see [29]–[31]). Readings were then downloaded to a PC by using the proprietary program ‘eTemperature’.

The room temperature was measured with a standard mercury thermometer, placed on a desk at approximately 100 cm from the participants. The temperature was recorded three times: 20, 47 and 66 minutes from the beginning of the experimental session. The average temperature measured with this procedure was of 27.5°C (± S.D. of 2.1°C).

The effects of the rTMS stimulation on the participant's perceived ownership of his/her hands were assessed by using two questionnaires (one for each hand) composed of 4 Likert scale questions each (see Appendix S1; cf. [32]).

### Procedure

The experiment was performed in a quiet room, with the participants being comfortably seated on a chair in front of a desk. Participants were asked to extend their arms, and rest their hands with their palms down on the desk at a distance of 50 cm from their trunk, and required to reduce to a minimum any body movement for all the duration of the experimental session. The temperature loggers were fixed to the back of the participants' hands with transpiring cotton tape. Temperature values were measured every five seconds for each hand.

The 24 volunteers were randomly assigned to receive rTMS to their left or right hemisphere. Each participant took part in two separate experimental sessions, one for each area stimulated. The two sessions were performed on different days and the order of sessions was counterbalanced across participants. A minimum interval of 24 hours occurred between the first and the second session (mean ± S. D. = 54 h±36.9 h, range 24–216 h).

**Figure 1:**
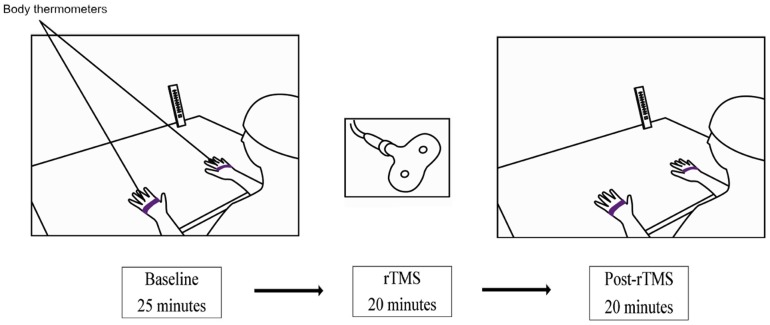
Setup and time course adopted in the present experiment.

Each experimental session lasted about 65 minutes, and comprised three phases: 1) baseline pre-TMS phase; 2) rTMS stimulation; and 3) post-rTMS phase (see Figure 1). In the baseline phase, which began after the experimenter had placed the temperature loggers on the participant's hands, the average temperature of the participant's hands was measured for 25 minutes. Hence, rTMS was delivered over PPC or S1 for a period of 20 minutes. Finally, in the last post-rTMS phase the average temperature of the participant's hands was measured again for a period of 20 minutes. At the end of the post-rTMS phase two participants in each group decided to leave the experiment and the remaining participants (N = 10 in each group) were presented with a questionnaire in order to evaluate their sense of ownership regarding their hands (see Appendix S1).

Low-frequency (1 Hz) off-line rTMS was delivered at a fixed intensity of 60% of the maximum stimulator output [33] using a Magstim Super Rapid magnetic stimulator (Magstim, Whitland, UK) and a figure-of-eight coil (70 mm diameter). TMS was applied over the PPC (left or right) and S1 (left or right). The rTMS stimulation sites were individually defined for each participant within the 10–20 electroencephalogram (EEG) coordinate system.

According to previous TMS studies [34], [35], the location of S1 was determined on an individual basis by moving the TMS coil posterior from the scalp location corresponding to the primary motor cortex that was found to produce maximal index finger movements in the contralateral hand. The resting motor threshold value was set to the stimulation level that elicited visible movements of the index finger in five of ten TMS pulses applied to the hand area. The posterior shift was repeated in 5-mm steps until TMS pulses at 120% of the motor threshold produced no visible finger movements and participants reported feeling no muscle twitches in response to the rTMS pulses. For PPC, the area of stimulation corresponded to 1 cm above the position P4 for the right PPC and to 1 cm above the position P3 for the left PPC in the 10∶20 EEG system [36], [37]. For both PPC and S1 rTMS, the coil was held tangentially to the scalp, with the handle pointing backwards at an angle of 45° from the midline. For each session, the correct site was marked on the participant's cap and the coil was positioned on that site for the duration of stimulation.

### Analysis

The average temperatures measured from both the temperature loggers during the last five minutes of the baseline condition (60 recordings) and the last 19 minutes (228 recordings) of the post-TMS condition were computed. The temperature data recorded during the twenty minutes of rTMS stimulation were not taken into consideration. These data were then submitted to a repeated-measures analysis of variance (ANOVA) with three within-subjects factors (Area: PPC, S1; Time: baseline, pre-rTMS, post-rTMS; Hand: right vs. left), and one between-subjects factor (targeted Hemisphere: right, left). Significant effects were assessed by Newman-Keuls post-hoc multiple comparisons.

The sums of the self-reported ratings of hand ownership from the four questions were also submitted to an ANOVA with two within-subjects factors (Area: PPC, S1; Hand: right, left), and one between-subjects factor (targeted Hemisphere: right, left). Note that, because question 1 referred to ownership, rather than disownership, the participants' responses to question 1 were subtracted from six, in order to make all of the responses homogenous in terms of the ownership effect that was measured (see Table 1, for the means of the responses given by the participants to each item individually).

**Table 1:** Mean response to each item of the questionnaire.

	LEFT HEMISPHERE															
	PPC								S1							
	Left hand				Right Hand				Left Hand				Right Hand			
	*Q. 1*	*Q. 2*	*Q. 3*	*Q. 4*	*Q. 1*	*Q. 2*	*Q. 3*	*Q. 4*	*Q. 1*	*Q. 2*	*Q. 3*	*Q. 4*	*Q. 1*	*Q. 2*	*Q. 3*	*Q. 4*
mean response	1.1	1.3	1	1.3	1.1	1	1	1.7	1.1	1.1	1	1.1	1.1	1	1.4	1.7

	RIGHT HEMISPHERE															
	PPC								S1							
	Left Hand				Right Hand				Left Hand				Right Hand			
	*Q. 1*	*Q. 2*	*Q. 3*	*Q. 4*	*Q. 1*	*Q. 2*	*Q. 3*	*Q. 4*	*Q. 1*	*Q. 2*	*Q. 3*	*Q. 4*	*Q. 1*	*Q. 2*	*Q. 3*	*Q. 4*
mean response	1.7	2	1.4	1.8	1.5	1.5	1.3	1.5	1.8	1.9	1.2	1.8	1.1	1	1.3	1.4

## Results

**Figure 2:**
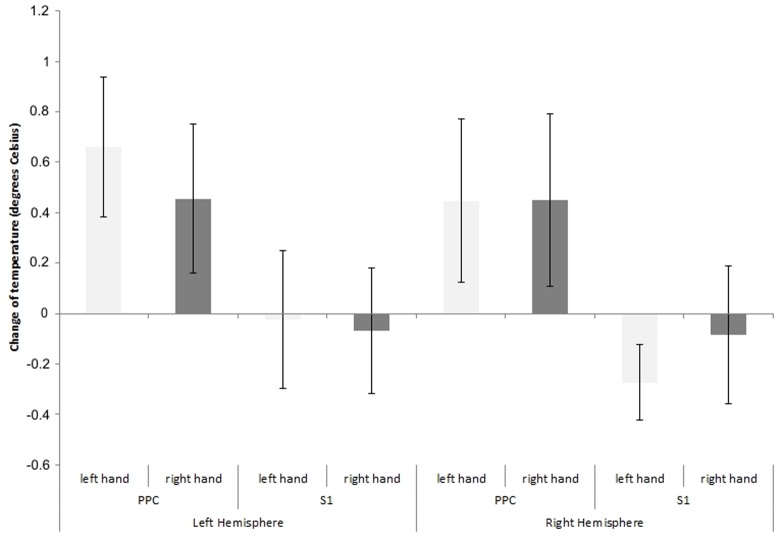
Mean decrease and standard errors of the mean (based on 288 observations per participant) in the temperature of the participants' hands (baseline minus post rTMS) as a function of the stimulated area (PPC = Posterior Parietal Cortex; S1 = Primary Somatosensory Cortex), and of the stimulated brain hemisphere. Temperature is reported using degrees Celsius. Positive values indicate higher temperatures measured in the baseline as compared to the post rTMS condition and viceversa.

**Figure 3:**
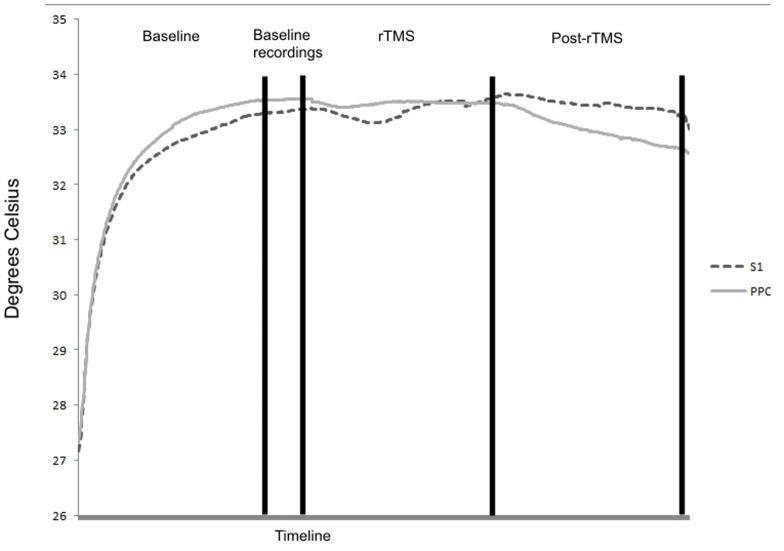
The mean temperatures recorded during all the experiment divided by site of rTMS stimulation.

Figure 2 shows the effects of rTMS (differences between pre and post rTMS values) delivered to the right and left PPC and S1 on temperature of the left and right hand. The temperature in both hands decreased (mean ±SE = : 0.5° Celsius ±0.06° Celsius) after rTMS of the PPC. rTMS to S1 had no effects (mean ± SE: −0.11° Celsius ±0.04° Celsius; see also Figure 3).

The ANOVA showed no significant main effects of Area [F(1, 22) = 0.21, p = .65, f^2^ = .99], Time [F(1, 22) = 1.76, p = .20, f^2^ = .92], Hand [F(1, 22) = 0.31, p = .58, f^2^ = .98], and Hemisphere [F(1, 22) = 0.03, p = .85, f^2^ = .99]. Importantly, the Area by Time interaction [F(1, 22) = 6.27, p = .02, f^2^ = .77] was significant. Newman-Keuls post-hoc comparisons showed that post rTMS temperature was lower than pre rTMS temperature for PPC stimulation (p = .04). No significant differences were reported between pre and post rTMS for S1 stimulation (p = .52), nor between S1 and PPC pre rTMS (p = 0.60). The Hemisphere by Area [F(1, 22) = 0.01, p = .91, f^2^ = .99], Hemisphere by Time [F(1, 22) = 0.17, p = .69, f^2^ = .99], Hemisphere by Hand [F(1, 22) = 1.70, p = .21, f^2^ = .92], Area by Time by Hemisphere [F(1, 22) = 0.00, p = .96, f^2^ = .99], Area by Hand by Hemisphere [F(1, 22) = 1.82, p = .19, f^2^ = .92], Area by Time by Hand [F(1, 22) = 2.59, p = .12, f^2^ = .89], and Area by Time by Hand by Hemisphere [F(1, 22) = 0.02, p = .90, f^2^ = .99] interactions were all not significant.

**Figure 4:**
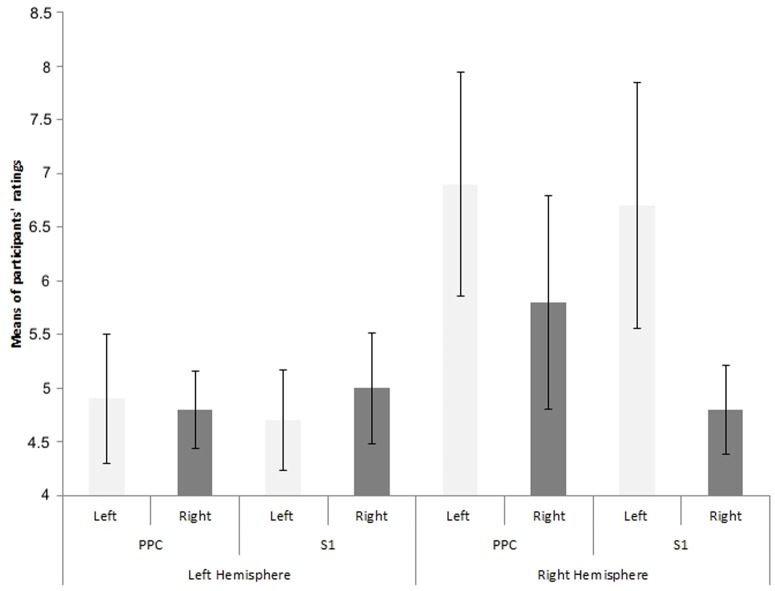
Questionnaire data. Means and standard error of the means of the individual sum of participants' ratings (ranging from 1 to 5) regarding the 4 statements of the questionnaire by stimulated area (PPC = Posterior Parietal Cortex; S1 = Primary Somatosensory Cortex), stimulated brain hemisphere, and participants' hand (left or right). Higher values represent larger sensations of body disownership regarding the specific body part investigated.

Figure 4 shows the effects of rTMS delivered to the right and left PPC and S1 on the participants' responses regarding their sense of ownership over the left and right hand. The ANOVA failed to reveal any significant main effect of Area [F(1,18) = 0.42, p = .52, f^2^ = .97], Hand F(1,18) = 1.32, p = .26, f^2^ = .93] or Hemisphere [F(1,18) = 3.52, p = .076, f^2^ = .83]. The Area by Hand [F(1,18) = 0.06, p = .80, f^2^ = .99], Area by Hemisphere [F(1,18) = 0.42, p = .52, f^2^ = .97], Hand by Hemisphere [F (1,18) = 1.72, p = .20, f^2^ = .91] and Area by Hand by Hemisphere [F (1,18) = 0.6, p = .45, f^2^ = .96] interactions were also not significant. That is, no changes in the participants' feelings of ownership regarding their right or left hand were reported after rTMS stimulation of either PPC or S1.

## Discussion

We hypothesised that rTMS over PPC would disrupt thermoregulation but rTMS over S1 would not. Our results clearly uphold this hypothesis – temperature of both hands was 0.5 degrees lower after rTMS over PPC, but was not significantly different after rTMS over S1. Our findings provide clear evidence that disruption of higher-order associative brain areas in humans can disrupt homeostatic control.

The results reported here offer additional empirical support for several studies that demonstrate that behavioral manipulations concerning the position of the body in space, as well as the sense of body ownership, can affect thermoregulatory control (see [5] for a review). Moreover, our findings are consistent with a recent model that suggests that a network of brain areas, comprising PPC, the premotor and the insular cortices, might play a crucial role in maintaining the integrity of the body at both the homeostatic (i.e., thermoregulation) and psychological (i.e., in terms of perception and the sense of body ownership) levels [5]. Within this structure, named the ‘body matrix’, multisensory information regarding the body and the space around it is constantly (and likely automatically) integrated. The results of the experiment reported here fully support this view by showing that a reversible functional interference of PPC, such as that provoked by rTMS, disrupts thermoregulatory control. The results of the present experiment therefore provide the first direct evidence that PPC might exert a top-down modulation of thermoregulatory control. It is, however, relevant to note that previous studies have shown that this brain area is part of a cortical/subcortical network involved in processing information regarding changes of body temperature [38]. That is, the PPC might be involved in both processing incoming signals regarding a variation of core body temperature, as well as in affecting the functioning of those efferent systems responsible for modulating such homeostatic variable.

It is important to highlight that our findings may also be in agreement with a large number of studies suggesting that higher order brain areas involved in integrating multisensory information, and in recalibrating information on the basis of different spatial frames of reference, also play a crucial role in supporting our representation of the body and our sense of body ownership [39]–[44]. Specifically, the present study extends these findings to suggest that these brain areas, and the PPC in particular, might also exert a top down influence on physiological mechanisms such as thermoregulatory control, related to the protection of the body integrity, a function strictly related to body ownership. This possibility has implications for chronic pain that extend beyond the condition of CRPS. For example, a recent discovery shows a spatially-defined disruption of tactile processing, similar to that observed in CRPS [1], in people with chronic unilateral back pain [45]. One might predict that, if the integration of spatial processing with autonomic control extends to the trunk, then autonomic dysfunction will also be observed in this patients' group. It is, however, worth mentioning that PPC is not a single general multimodal processor, but has distinct functional subregions in the monkey and human (see [46] for a review). Therefore, further research might also target which specific areas of the PPC are involved in thermoregulatory control.

From a neurological standpoint, lesions of the anterior parietal lobe, including S1, may bring about a contralateral deficit of temperature sensation, together with a deficit of touch, pain, tactile discrimination and stereognosis [47], [48]. Damage to the superior parietal lobule of the PPC affects mainly tactile discrimination and stereognosis [49], and sensorimotor integration [50]. When the inferior parietal lobule of the PPC is damaged, deficits such as tactile agnosia may be found [51]. Although the majority of studies do not report hemispheric asymmetries [52], there is evidence that somatosensory deficits are more frequent after damage to the right hemisphere than to the left [53], which probably reflects a main role of the right hemisphere for somatosensory attention and perception, for both the left contralateral, and the right ipsilateral, sides of the body [54]–[56]. This hemispheric asymmetry may be related to the fact that awareness of somatosensory events involves their coding in spatial reference frames, an operation for which the right hemisphere is specialized [57]–[60]. Conversely, more basic processes supported by the body matrix [5], such as thermoregulation, and, as recent evidence suggests - immune regulation [4] - might be more symmetrically-distributed across the two cerebral hemispheres. This suggestion would appear to be confirmed by the lack of differences found in the present experiment between rTMS over the right and left PPC on thermoregulatory control.

In the present experiment no differences between the two hands were reported in the decrease of temperature elicited by rTMS over the PPC. By contrast, behavioral experiments have shown body district-specific effects on thermoregulatory control ([1], [5], though see [61], for a lack of body-district specific effects when a full body illusion is elicited). Here one might speculate on whether this lack of more specific effects might be due to the fact that the body matrix likely comprises both motor and perceptual systems [5]. That is, body district-specific effects might be related to the functioning of the motor (i.e., the premotor cortex) rather than to the more perceptual components of the body matrix. Note in fact that one of the ultimate purposes of this cortical network is to protect the body integrity by performing accurate movements. Moreover, there is ample evidence showing that representations posterior to SI, such as those in SII receive inputs from both body sides [62], [63], (cf. [64], for a review on the presence of neurons with bilateral receptive fields also within S1 in animals).

As far as the point of the lack of body specific effects is concerned, it is worth noting here that behavioural experiments where the modulation of thermoregulatory control was confined to a specific body district have adopted the paradigm of the rubber hand illusion [1], [5]. Within such paradigm the participants who are exposed to visuo-tactile synchronous information, start to perceive that a fake hand is part of their own body. One might then argue that, despite of the body specificity of the effect that this illusion exerts on thermoregulatory control, no motor components seems to be involved in generating the phenomenon (in fact the participants keep their arm still). However, one should consider that changes in the perception of where or body is in space, likely involves the access to the body schema, where information regarding the position of the body is continuously updated in order to program accurate movements towards external space. One might therefore speculate that the motor component of the body matrix is activated also under such condition of stimulus presentation. As far as this concept is concerned, it is important to notice that studies on the rubber hand illusion have shown that changes in the perceived position of one's body often occur without any alteration of the participant's sense of body ownership [65].

It should also be considered, that the results reported in the present study might not be due to the fact that interference over PPC disrupts the activity of a network involved in supporting a representation of the body and the sense of body ownership (note in fact that we did not find any changes in body ownership following the stimulation of PPC, see also below). By contrast, they might result from a disruption in the functioning of a network involved in directing attention towards external space. In fact, a number of studies on brain damaged patients, as well as on neurological unimpaired participants, have clearly shown that the PPC is part of a neural circuit involved in the deployment of spatial and selective attention in humans [55], (see also [66], [67] for TMS studies). It is however important to note, that no study has so far directly investigated the role of attentional shifts on thermoregulatory control (though see [8] for the effect of prism adaptation – a procedure that has been shown to modulate spatial attention mechanisms – on thermoregulatory control in patients affected by chronic regional pain syndrome). This topics certainly deserves to be addressed in the near future.

The results from the questionnaire, showed that interference with the functioning of PPC or S1 does not result into an impairment of the participants' sense of body ownership. This result might be taken to suggest that rTMS over PPC might affect in different fashions, physiological and cognitive functions. However, before drawing any conclusion on the basis of this result, one should consider that the participants in our study were not neurologically damaged patients and they certainly knew that the arm in front of them was their own arm. That is, the effect of rTMS might be not as powerful to overcome people's explicit beliefs and expectations regarding their body ownership, but sufficient to alter their (implicitly-controlled) physiological responses. It certainly remains possible that different effects of rTMS might be expected when more robust measuring tools thought to be affected by illusions of body ownership, such as the spatial localization of a person's own body parts, are used instead.

In conclusion, the results of the present experiment show that higher order cortical areas such as PPC might play an important role in sustaining and/or modulating lower level physiological functions such as thermoregulatory control.

## Supporting Information

Appendix S1 Ownership of right/left hand questionnaire.
(DOCX)

## References

[1] Moseley GL, Olthof N, Venema A, Don S, Wijers M, et al (2008) Psychologically induced cooling of a specific body part caused by the illusory ownership of an artificial counterpart. Proceedings of the National Academy of Science USA 105: 13169–13173. 10.1073/pnas.0803768105 PMC2529116 18725630

[2] Tsakiris M, Tajadura-Jiménez AT, Costantini M (2011) Just a heartbeat away from one's body: interoceptive sensitivity predicts malleability of body-representations. Proceedings of the Royal Society Biological Sciences 278: 2470–2476. 21208964 10.1098/rspb.2010.2547 PMC3125630

[3] Kammers MP, Rose K, Haggard P (2011) Feeling numb: Temperature, but not thermal pain, modulates feeling of body ownership. Neuropsychologia 49: 1316–1321. 21354190 10.1016/j.neuropsychologia.2011.02.039

[4] Barnsley N, McAuley J, Mohan R, Dey A, Thomas P, et al (2011) The rubber hand illusion increases histamine reactivity in the real arm. Current Biology 21: R945–R946. 22153159 10.1016/j.cub.2011.10.039

[5] Moseley GL, Gallace A, Spence C (2012) Bodily illusion in health and disease: physiological and clinical perspectives and the concept of a cortical body matrix. Neuroscience & Biobehavioural Reviews 36: 34–46. 10.1016/j.neubiorev.2011.03.013 21477616

[6] Moseley GL, Gallace A, Spence C (2009) Space-based, but not arm-based, shift in tactile processing in complex regional pain syndrome and its relationship to cooling of the affected limb. Brain 132: 3142–3151. 19752177 10.1093/brain/awp224

[7] Moseley GL, Gallace A, Iannetti GD (2012) Spatially defined modulation of skin temperature and hand ownership of both hands in patients with unilateral complex regional pain syndrome. Brain 135: 3676–3686. 23250885 10.1093/brain/aws297

[8] Moseley GL, Gallace A, di Pietro F, Spence C, Iannetti GD (2013) Limb-specific autonomic dysfunction in complex regional pain syndrome modulated by wearing prism glasses. Pain doi: pii: S0304-3959(13)00394-1. 10.1016/j.pain.2013.07.026 10.1016/j.pain.2013.07.026 23886518

[9] Petchkrua W, Weiss DJ, Patel RR (2000) Reassessment of the incidence of complex regional pain syndrome type 1 following stroke. Neurorehabilitation and Neural Repair 14: 59–63. 11228950 10.1177/154596830001400107

[10] Marinus J, Moseley GL, Birklein F, Baron R, Maihöfner C, et al (2011) Clinical features and pathophysiology of complex regional pain syndrome. Lancet Neurology 10: 637–648. 21683929 10.1016/S1474-4422(11)70106-5 PMC5511749

[11] Acerra NE, Souvlis T, Moseley GL (2007) Stroke, complex regional pain syndrome and phantom limb pain: Can Commonalities Direct Future Management? Journal of Rehabilitation Medicine 39: 109–114. 17351691 10.2340/16501977-0027

[12] Melzack R (1990) Phantom limbs and the concept of a neuromatrix. Trends in Neurosciences 13: 88–92. 1691874 10.1016/0166-2236(90)90179-e

[13] Ehrsson HH, Holmes NP, Passingham RE (2005) Touching a rubber hand: feeling of body ownership is associated with activity in multisensory brain areas. Journal of Neuroscience 25: 10564–10573. 16280594 10.1523/JNEUROSCI.0800-05.2005 PMC1395356

[14] Ellis LB, Weiss S (1936) Vasomotor disturbance and oedema associated with cerebral hemiplegia. Archives of Neurology & Psychiatry 35: 362–372.

[15] Wanklyn P, Isley DW, Greenstein D, Hampton IFG, Roper TA, et al (1994) The cold hemiplegic arm. Stroke 25: 1765–1770. 8073457 10.1161/01.str.25.9.1765

[16] Naver H, Blomstrand C, Ekholm S, Jensen C, Karlsson T, et al (1995) Autonomic and thermal sensory symptoms and dysfunction after stroke. Stroke; A journal of cerebral circulation 26: 1379–1385. 10.1161/01.str.26.8.1379 7631341

[17] Riedl B, Beckmann T, Neundorfer B, Handwerker HO, Birklein F (2001) Autonomic failure after stroke–is it indicative for pathophysiology of complex regional pain syndrome? Acta Neurologica Scandinavica 103: 27–34. 11153885 10.1034/j.1600-0404.2001.00139.x

[18] Ishii K, Ohkoshi N, Tamaoka A, Mizusawa H, Shoji S (1996) Pseudoradicular sensory impairment caused by parietal lesions. Report of two cases. Rinsho Shinkeigaku. Clinical Neurology 36: 951–956. 8958747

[19] Satoh M, Terada S, Onouchi K, Takeda K, Kuzuhara S (2002) Somatosensory and skin temperature disturbances caused by infarction of the postcentral gyrus: a case report. Journal of Neurology 249: 1404–1408. 12382157 10.1007/s00415-002-0853-7

[20] De Luca B, Monda M, Amaro S, Pellicano MP, Ciuffi LA (1989) Thermogenic changes following frontal neocortex stimulation. Brain Research Bulletin 22: 1003–1007. 2790492 10.1016/0361-9230(89)90012-9

[21] Robertson EM, Théoret H, Pascual-Leone A (2003) Studies in cognition: The problems solved and created by transcranial magnetic stimulation. Journal of Cognitive Neuroscience 15: 948–960. 14614806 10.1162/089892903770007344

[22] Hallett M (2007) Transcranial magnetic stimulation: A primer. Neuron 55: 187–199. 17640522 10.1016/j.neuron.2007.06.026

[23] Miniussi C, Ruzzoli M, Walsh V (2010) The mechanism of transcranial magnetic stimulation in cognition. Cortex 46: 128–130. 19356747 10.1016/j.cortex.2009.03.004

[24] Walsh V, Cowey A (2000) Transcranial magnetic stimulation and cognitive neuroscience. Nature Reviews Neuroscience 1: 73–79. 11252771 10.1038/35036239

[25] Fregni F, Pascual-Leone A (2007) Technology insight: Noninvasive brain stimulation in neurology-perspectives on the therapeutic potential of rTMS and tDCS. Nature Clinical Practice Neurology 3: 383–393. 10.1038/ncpneuro0530 17611487

[26] Vallar G, Ronchi R (2009) Somatoparaphrenia: A body delusion. A review of the neuropsychological literature. Experimental Brain Research 192: 533–551. 18813916 10.1007/s00221-008-1562-y

[27] Oldfield RC (1971) The assessment and analysis of handedness: the Edinburgh inventory. Neuropsychologia 9: 97–113. 5146491 10.1016/0028-3932(71)90067-4

[28] Rossi S, Hallett M, Rossini PM, Pascual-Leone A (2009) Safety, ethical considerations, and application guidelines for the use of transcranial magnetic stimulation in clinical practice and research. Clinical Neurophysiology 120: 2008–2039. 19833552 10.1016/j.clinph.2009.08.016 PMC3260536

[29] Harper Smith AD, Crabtree DR, Bilzon JLJ, Walsh NP (2010) The validity of wireless iButtons and thermistors for human skin temperature measurement. Physiological Measurement 31: 95–114. 19940348 10.1088/0967-3334/31/1/007

[30] Hasselberg MJ, McMahon J, Parker K The validity, reliability, and utility of the ibutton for measurement of body temperature circadian rhythms in sleep/wake research. Sleep Medicine In press. 10.1016/j.sleep.2010.12.011 21470909

[31] van Marken Lichtenbelt WD, Daanen HAM, Wouters L, Fronczek R, Raymann RJEM, et al (2006) Evaluation of wireless determination of skin temperature using iButtons. Physiology & Behavior 88: 489–497. 16797616 10.1016/j.physbeh.2006.04.026

[32] Botvinick M, Cohen J (1998) Rubber hands “feel” touch that eyes see. Nature 391: 756. 9486643 10.1038/35784

[33] Cappelletti M, Barth H, Fregni F, Spelke ES, Pascual-Leone A (2007) rTMS over the intraparietal sulcus disrupts numerosity processing. Experimental Brain Research 179: 631–642. 17216413 10.1007/s00221-006-0820-0 PMC2567820

[34] Fiorio M, Haggard P (2005) Viewing the body prepares the brain for touch: effects of tms over somatosensory cortex. European Journal of Neuroscience 22: 773–777. 16101759 10.1111/j.1460-9568.2005.04267.x

[35] Oliveira FTP, Diedrichsen J, Verstynen T, Duque J, Ivry RB (2010) Transcranial magnetic stimulation of posterior parietal cortex affects decisions of hand choice. Proceedings of the National Academy of Sciences 107: 17751–17756. 10.1073/pnas.1006223107 PMC2955129 20876098

[36] Herwig U, Satrapi P, Schonfeldt-Lecuona C (2003) Using the international 10–20 EEG system for positioning of transcranial magnetic stimulation. Brain Topography 16: 95–99. 14977202 10.1023/b:brat.0000006333.93597.9d

[37] Okamoto M, Dan H, Sakamoto K, Takeo K, Shimizu K, et al (2004) Three-dimensional probabilistic anatomical cranio-cerebral correlation via the international 10–20 system oriented for transcranial functional brain mapping. Neuroimage 21: 99–111. 14741647 10.1016/j.neuroimage.2003.08.026

[38] Egan GF, Johnson J, Farrell M, McAllen R, Zamarripa F, et al (2005) Cortical, thalamic, and hypothalamic responses to cooling and warming the skin in awake humans: a positron-emission tomography study. Proceedings of the National Academy of Sciences 102: 5262–5267. 10.1073/pnas.0409753102 PMC555992 15793009

[39] Giummarra MJ, Gibson SJ, Georgiou-Karistianis N, Bradshaw JL (2008) Mechanisms underlying embodiment, disembodiment and loss of embodiment. Neuroscience and Biobehavioural Reviews 32: 143–160. 10.1016/j.neubiorev.2007.07.001 17707508

[40] Longo MR, Schüür F, Kammers MPM, Tsakiris M, Haggard P (2008) What is embodiment? a psychometric approach. Cognition 107: 978–998. 18262508 10.1016/j.cognition.2007.12.004

[41] Makin TR, Holmes NP, Ehrsson HH (2008) On the other hand: Dummy hands and peripersonal space. Behavioural Brain Research 191: 1–10. 18423906 10.1016/j.bbr.2008.02.041

[42] Tsakiris M (2010) My body in the brain: A neurocognitive model of body-ownership. Neuropsychologia 48: 703–712. 19819247 10.1016/j.neuropsychologia.2009.09.034

[43] Tsakiris M, Costantini M, Haggard P (2008) The role of the right temporo-parietal junction in maintaining a coherent sense of one's body. Neuropsychologia 46: 3014–3018. 18601939 10.1016/j.neuropsychologia.2008.06.004

[44] Tsakiris M, Longo MR, Haggard P (2010) Having a body versus moving your body: neural signatures of agency and body-ownership. Neuropsychologia 48: 2740–2749. 20510255 10.1016/j.neuropsychologia.2010.05.021

[45] Moseley GL, Gallagher L, Gallace A (2012) Neglect-like tactile dysfunction in chronic back pain. Neurology 79: 327–332. 22744662 10.1212/WNL.0b013e318260cba2

[46] Grefkes C, Fink GR (2005) The functional organization of the intraparietal sulcus in humans and monkeys. Journal of Anatomy 207: 3–17. 16011542 10.1111/j.1469-7580.2005.00426.x PMC1571496

[47] Bassetti C, Bugousslavsky J, Regli F (1993) Sensory syndromes in parietal stroke. Neurology 43: 1942–1949. 8413950 10.1212/wnl.43.10.1942

[48] Kim JS (2007) Patterns of sensory abnormality in cortical stroke. evidence for a dichotomized sensory system. Neurology 68: 174–180. 17224568 10.1212/01.wnl.0000251298.12763.9b

[49] Freund H-J (2003) Somatosensory and motor disturbances in patients with parietal lobe lesions. Advances in Neurology 93: 179–193. 12894408

[50] Wolpert DM, Goodbody SJ, Husain M (1998) Maintaining internal representations: The role of the human superior parietal lobe. Nature Neuroscience 1: 529–533. 10196553 10.1038/2245

[51] Reed CL, Caselli RJ, Farah MJ (1996) Tactile agnosia. Underlying impairment and implications for normal tactile object recognition. Brain 119: 875–888. 8673499 10.1093/brain/119.3.875

[52] Dijkerman HC, de Haan EH (2007) Somatosensory processes subserving perception and action. Behavioral and Brain Sciences 30: 189–201. 17705910 10.1017/S0140525X07001392

[53] Sterzi R, Bottini G, Celani MG, Righetti E, Lamassa M, et al (1993) Hemianopia, hemianaesthesia, and hemiplegia after left and right hemisphere damage: A hemispheric difference. Journal of Neurology Neurosurgery and Psychiatry 56: 308–310. 10.1136/jnnp.56.3.308 PMC1014869 8459249

[54] Bottini G, Paulesu E, Gandola M, Loffredo S, Scarpa P, et al (2005) Left caloric vestibular stimulation ameliorates right hemianesthesia. Neurology 65: 1278–1283. 16247057 10.1212/01.wnl.0000182398.14088.e8

[55] Pardo JV, Fox PT, Raichle ME (1991) Localization of a human system for sustained attention by positron emission tomography. Nature 349: 61–64. 1985266 10.1038/349061a0

[56] Vallar G (2007) A hemispheric asymmetry in somatosensory processing. Behavioral and Brain Sciences 30: 223–224.

[57] Gallace A, Spence C (2008) The cognitive and neural correlates of “tactile consciousness”: a multisensory perspective. Consciousness and Cognition 17: 370–407. 17398116 10.1016/j.concog.2007.01.005

[58] Gallace A, Spence C (2010) The role of the somatosensory cortex in the awareness of tactile information. Psyche 16: 30–67.

[59] Kitazawa S (2002) Where conscious sensation takes place. Consciousness and Cognition 11: 475–477. 12435379 10.1016/s1053-8100(02)00031-4

[60] Vallar G (1997) Spatial frames of reference and somatosensory processing: a neuropsychological perspective. In N. Burgess, K. J. Jeffery & J. O'Keefe (Eds.), The Hippocampal and Parietal Foundations of Spatial Cognition (pp. 33–49). Oxford: Oxford University Press. 10.1098/rstb.1997.0126 PMC1692053 9368928

[61] Salomon R, Lim M, Pfeiffer C, Gassert R, Blanke O (2013) Full body illusion is associated with widespread skin temperature reduction. Frontiers in Behavioral Neurosciences 7: 65 doi:10.3389/fnbeh.2013.00065 10.3389/fnbeh.2013.00065 PMC3724056 23898244

[62] Blatow M, Nennig E, Durst A, Sartor K, Stippich C (2007) fMRI reflects functional connectivity of human somatosensory cortex. Neuroimage 37: 927–936. 17629500 10.1016/j.neuroimage.2007.05.038

[63] Ferretti A, Babiloni C, Gratta CD, Caulo M, Tartaro A, et al (2003) Functional topography of the secondary somatosensory cortex for nonpainful and painful stimuli: an fMRI study. Neuroimage 20: 1625–1638. 14642473 10.1016/j.neuroimage.2003.07.004

[64] Iwamura Y (2000) Bilateral receptive field neurons and callosal connections in the somatosensory cortex. Philosophical Transactions of the Royal Society of London B 355: 267–273. 10.1098/rstb.2000.0563 PMC1692728 10724460

[65] Rohde M, Di Luca M, Ernst MO (2011) The Rubber Hand Illusion: feeling of ownership and proprioceptive drift do not go hand in hand. PloS one 6 (6) e21659. 21738756 10.1371/journal.pone.0021659 PMC3125296

[66] Blankenburg F, Ruff CC, Bestmann S, Bjoertomt O, Josephs O, et al (2010) Studying the role of human parietal cortex in visuospatial attention with concurrent TMS-fMRI. Cerebral Cortex 20: 2702–2711. 20176690 10.1093/cercor/bhq015 PMC2951847

[67] Hung J, Driver J, Walsh V (2005) Visual selection and posterior parietal cortex: effects of repetitive transcranial magnetic stimulation on partial report analyzed by Bundesen's theory of visual attention. Journal of Neuroscience 25: 9602–9612. 16237165 10.1523/JNEUROSCI.0879-05.2005 PMC6725743

