# Supplementary material for: Temporary Interference over the Posterior Parietal Cortices Disrupts Thermoregulatory Control in Humans
Source: PLoS One. 2014 Mar 12;9(3):e88209. doi: 10.1371/journal.pone.0088209 (PMC3951183; doi:10.1371/journal.pone.0088209)
Supplement: Appendix S1 — Ownership of right/left hand questionnaire. (DOCX) [file pone.0088209.s001.docx]

Appendix 1

**OWNERSHIP OF RIGHT/LEFT HAND QUESTIONNAIRE**

Mark the level of agreement to the following statements using the scale below:

**1**: not at all

**2**: not much

**3**: slightly

**4**: very much

**5**: totally

|  | **1** | **2** | **3** | **4** | **5** |
| --- | --- | --- | --- | --- | --- |
| 1) I feel that the right/left hand belongs to me |  |  |  |  |  |
| 2) I feel that the right/left hand belongs to me less than the other |  |  |  |  |  |
| 3) My right/left hand seems to be different in color, shape and size than usual |  |  |  |  |  |
| 4) I feel particular sensations (tingling and numbness, etc.) on my right/left hand |  |  |  |  |  |
